# The Highly Conserved *Escherichia coli* Transcription Factor YhaJ Regulates Aromatic Compound Degradation

**DOI:** 10.3389/fmicb.2016.01490

**Published:** 2016-09-22

**Authors:** Noa Palevsky, Benjamin Shemer, James P. R. Connolly, Shimshon Belkin

**Affiliations:** ^1^Institute of Life Sciences, Hebrew University of JerusalemJerusalem, Israel; ^2^College of Medical, Veterinary and Life Sciences, University of GlasgowGlasgow, UK

**Keywords:** *Escherichia coli*, dinitrotoluene, trinitrotoluene, *yqjF*, *YhaJ*, transcriptional regulation, bioreporters

## Abstract

The aromatic compound 2,4-dinitrotoluene (DNT), a common impurity in 2,4,6-trinitrotoluene (TNT) production, has been suggested as a tracer for the presence of TNT-based landmines due to its stability and high volatility. We have previously described an *Escherichia coli* bioreporter capable of detecting the presence of DNT vapors, harboring a fusion of the *yqjF* gene promoter to a reporter element. However, the DNT metabolite which is the direct inducer of *yqjF*, has not yet been identified, nor has the regulatory mechanism of the induction been clarified. We demonstrate here that the YhaJ protein, a member of the LysR type family, acts as a transcriptional regulator of *yqjF* activation, as well as of a panel of additional *E. coli* genes. This group of genes share a common sequence motif in their promoters, which is suggested here as a putative YhaJ-box. In addition, we have linked YhaJ to the regulation of quinol-like compound degradation in the cell, and identified *yhaK* as playing a role in the degradation of DNT.

## Introduction

We have previously described (Yagur-Kroll et al., 2014, 2015) an *Escherichia coli* bioreporter strain engineered to sensitively detect traces of 2,4,6-trinitrotoluene (TNT) and 2,4-dinitrotoluene (DNT), both signature compounds of most buried landmines and other explosive devices. The sensing element in this construct was the promoter of *yqjF*, a gene of unknown function. This gene promoter was induced in a distinct dose-dependent manner by both DNT and TNT, but its activation kinetics indicated that it was not activated directly by either of these compounds but rather by their yet unidentified metabolites. Neither the mechanism of activation nor its regulation have been described.

Several transcriptional regulatory protein families are involved in the degradation of aromatic compounds in bacteria, differing both in their structure and in the activation or repression mechanisms. Among these are representatives of the AraC, GntR, XylR, FNR, IclR, and LysR protein families (Tropel and Van Der Meer, 2004). Members of the latter group, LysR-type transcriptional regulators (LTTRs), are characterized by an N-terminal DNA-binding helix-turn-helix motif and a C-terminal co-inducer-binding domain (Schell, 1993). According to Tropel and Van Der Meer (2004), all LTTRs involved in aromatic compounds degradation pathways act as transcriptional activators with the co-inducer being either the degraded compound or an intermediate in the degradation pathway. In many cases, LTTRs and their regulated genes are divergently transcribed, with the LTTR having an auto-regulation mechanism (Maddocks and Oyston, 2008).

Two transcriptional factors in bacteria have previously been found to bind 2,4-DNT. These are DntR from *Burkholderia* sp. (Suen and Spain, 1993) and NtdR in *Acidovorax* sp. strain JS42 (Lessner et al., 2003). Both are LTTRs, and their amino acid sequence is 97% identical. Both regulators are thought to have recently diverged from the naphthalene degradation regulator NagR of *Ralstonia* sp. U2. DntR is the transcription factor that activates the oxidative degradation of 2,4-DNT, and NtdR activates the expression of genes involved in 2-nitrotoluene degradation in response to 2,4-DNT and to several other nitroaromatic compounds (Ju et al., 2009).

In the study reported herein we have endeavored to identify and describe the regulatory mechanism responsible for *yqjF* induction by DNT; the results described below have led us to propose that YhaJ, an emerging member of the LysR type family, is the transcriptional regulator of *yqjF*, as well as several other genes.

## Materials and Methods

### Chemicals

All chemicals used in this work were of the highest analytical grade and were purchased from Sigma-Aldrich. All hydrophilic chemicals were dissolved in double-distilled water while the hydrophobic ones were dissolved in 96% ethanol.

### Bacterial Strains

*Escherichia coli* K12 strain DH5α was used as a host for the construction of all plasmids. *E. coli* K12 strain MG1655 was used as the bioreporter platform for the chemicals’ screen and its chromosomal DNA was used as a template for all PCR reactions. All JW strains were taken from the “Keio collection” single-gene knockout mutants’ library (Baba et al., 2006). Plasmid pcp20 was employed for extraction of kanamycin resistance from these strains. Strain BL21 (DE3) was used as a host cell for overexpression of the YhaJ:his tagged protein. All strains and plasmids used in the course of the current study are listed in **Table 1**.

**Table 1:** *Escherichia coli* strains and plasmids employed in this study.

	Description	Reference
**Strains**		
BL21(DE3)	huA2 [Ion] ompT gal (X DE3) [dcm] AhsdS	Studier and Moffatt, 1986
	X DE3 = X sBamHIo AEcoRI-B int::(lad::PlacUV5::T7 genel) i21 Anin5	
MG1655	F∼X∼ ilvG-rfb-50 rph-1	Blattner et al., 1997
DH5a	F-endAl hsdR17 (rk-,mk+) supE44 thi-1 XrecAl gyrA96 relAl deoR A(lacZYA-argF)-U169 < J)80dlacZAM15	Grant et al., 1990
BW25113	Keio collection wild-type *E. coli* K-12 strain	Baba et al., 2006
AyhaJ	BW25113 with yhaJ deletion	Baba et al., 2006
AyhaK	BW25113 with yhaK deletion	Baba et al., 2006
**Plasmids**		
pCP20	Red recombinase expression plasmid	Cherepanov and Wackernagel, 1995
**Plasmids**		
yqjF::GPF	pUA66 derivative carrying yqjF::GPFmut2	Zaslaver et al., 2006
ybiJ::GPF	pUA139 derivative carrying ybiJ::GPFmut2	Zaslaver et al., 2006
yhhW::GFP	pUA139 derivative carrying yhhW::GPFmut2	Zaslaver et al., 2006
ygiD::GFP	pUA139 derivative carrying ygiD::GPFmut2	Zaslaver et al., 2006
ycep::GFP	pUA139 derivative carrying ycep::GPFmut2	Zaslaver et al., 2006
yhaK::GFP	pUA66 derivative carrying yhaK::GPFmut2	Zaslaver et al., 2006
yqjFwt::lux	pBR322 derivative carrying yqjFwt::luxCDABE	Yagur-Kroll et al., 2014
yqjFmutl::lux	yqjFwt::lux derivative carrying point mutation C146T ^∗^	This work
yqjFmut2::lux	yqjFwt::lux derivative carrying point mutations T138C, T137C ^∗^	This work
yqjFmut3::lux	yqjFwt::lux derivative carrying point mutation A143T ^∗^	This work
yqjFmut4:lux	yqjFwt::lux derivative carrying point mutations A144G, A143G, T142A, T141A, T140A, G133C ^∗^	This work
ybiJmutl::GPF	ybiJ:GFP derivative carrying a point mutation C120T^∗^	This work
ybiJmut2::GPF	ybiJ:GFP derivative carrying point mutations T112C, T111C^∗^	This work
ygiDmutl::GFP	ybiJ:GFP derivative carrying a point mutation C50T^∗^	This work
ygiDmut2::GFP	ybiJ:GFP derivative carrying point mutations T42C, T41C^∗^	This work
yhhWmut 1::GFP	yhhw::GFP derivative carrying a point mutation C99T^∗^	This work
yhhWmut2::GFP	yhhw::GFP derivative carrying point mutations T91C, T92C^∗^	This work
yhaKmutl::GFP	yhaK::GFP derivative carrying a point mutation C92T^∗^	This work
yhaKmut2::GFP	yhhw::GFP derivative carrying point mutations T84C, T83C^∗^	This work
yqjFwt::lux’.yhaJ: :yhaJ	pBR322 derivative carrying yqjFwt::luxCDABE and yhaJ::yhaJ	This work
pET-28b::yhaJ-his	pET-28b derivative carrying yhaJ-his, for yhaJ expression and purification	Connolly et al., 2016


*^∗^The mutation position is upstream relative to the ATG of each gene.*

### Plasmid Construction

Promoter segments of yqjF harboring different sets of mutations were cloned (**Table 1**) into plasmid pBR2TTS::luxCDABE (Yagur-Kroll et al., 2014). Two of these sets were also inserted into the promoters of yhaK, ybiJ, ygiD and yhhW, and cloned into plasmids pUA66 and pUA139 (Zaslaver et al., 2006). Overlapping PCR was used to insert the mutations into the desired locations in the promoter regions. Two sets of amplification were performed to generate two overlapping fragments harboring the required mutation set for each promoter, with one primer harboring the restriction enzyme site at the end of the promoter sequence and the other harboring the mutation set in its middle. Next, the two PCR fragments were subjected to 15 cycles of PCR without primers or template, followed by 22 cycles of PCR in the presence of the end primers to obtain a sufficient amount of the promoter sequence. A List of primers used in this study is presented in **Supplementary Table S1**.

### Purification of Recombinant YhaJ

The *yhaJ* nucleotide sequence was cloned into plasmid pET-28b (N-terminal 6xHistidine tag) using BamHI (forward) and HindIII (reverse) in-frame restriction flanks. Positive clones were confirmed by sequencing, and *E. coli* BL21 DE3 cells were transformed with this plasmid for overexpression with IPTG induction. A 5 ml overnight culture in LB was used to inoculate 1 l of LB in a 2.5 l ruffled Erlenmeyer flask and cultures were grown at 37°C and 200 rpm until they reached an OD_600_ of 0.6. Cultures were then induced with 1 mM IPTG and incubated at 15°C overnight with shaking. Cells were harvested by centrifugation, resuspended in wash buffer (200 mM NaCl, 50 mM Tris, 40 mM imidazole, 10% glycerol) and split with a French Press. The supernatant was filtered and YhaJ was purified by Ni-affinity chromatography (AKTA-prime, GE Healthcare Life Sciences) according to the manufacturer’s specifications. Further purification was carried out using size-exclusion chromatography employing an AKTA-prime equipped with Superdex S200 column. Size and purity of YhaJ were verified by SDS-PAGE. Concentration of the purified protein was determined by Nanodrop 2000 (Thermo Scientific).

### Electrophoretic Mobility Shift Assay (EMSA)

Electrophoretic mobility shift assay (EMSA) was carried out using the DIG Gel Shift Kit (Roche, Mannheim, Germany) according the manufacturer’s specifications with minor modifications. Approximately 300 bp DNA probes corresponding to desired promoter regions were amplified by PCR using primers specific to the regions of interest. DNA probes were purified with a PCR purification kit (Qiagen) and quantified by Nanodrop 2000. Probes were labeled with ddUTP-11-DIG and diluted to a concentration of approximately 0.4 ng/μl for use in binding reactions. Reactions were carried out for 20 min at 30°C using 0.5 μM of recombinant YhaJ for binding. Competition assays to test for binding specificity used a 20-fold excess of unlabeled specific or non-specific probe in the reaction mix. A fragment of the *kan* gene was used as a non-specific control probe. Reactions were separated on 6% DNA retardation gels (Invitrogen) ran at 80 volts for 100 min, and transferred to a positively charged nylon membrane (Roche) using the NOVEX system (Invitrogen). Transferred membranes were then probed with AP-conjugated anti-DIG antibody (Roche) and developed according to the manufacturer’s specifications. Assays were repeated three times for confirmation of results.

### Promoter::GFPmut2 Library Screening for Hydroquinone-Inducible Gene Promoters

A library of approximately 2,000 reporter strains (Zaslaver et al., 2006), each bearing a low-copy plasmid with a different promoter controlling a GFP gene (GFPmut2) expressed in *E. coli* K12 strain MG1655, was kindly provided by Prof. Uri Alon from the Department of Molecular Cell Biology at the Weizmann Institute of Science, Rehovot, Israel. Prior to the assay the library was replicated into 96 well plates with 175 μl TGA [10 g/l Bacto Tryptone, 5 g/L NaCl, 2 g/l D-(+)glucose, 11.9 g/l HEPES (4-(2-hydroxyethyl)-1-piperazineethanesulfonic acid, pH 7.0] with kanamycin (30 μg/ml), and grown overnight. Culture aliquots (10 μl) were transferred into two black 96-well microtiter plates, each well already containing either 90 μl TGA with 100 mg/l hydroquinone or just 90 μl TGA for the control. Fluorescence (excitation 485 nm, emission 535 nm) was measured in an Infinite M200 PRO (Tecan) plate reader, at 40 min intervals for 10 h.

### Measuring the Response of Reporter Strains to 2,4-DNT and Related Compounds

Response of bioluminescent and fluorescent reporter strains to the tested chemicals was assayed following overnight growth (37°C, 200 rpm) in 2 mL LB (luminescent reporter strains) or TGA (fluorescent reporter strains) with the respective antibiotic, dilution 100-fold in 3 mL of the same media, and regrowth under the same conditions to an OD_600_ of 0.15. Culture aliquots (50 μl) were transferred into a 96-well microtiter plate, each well already containing 50 μl of a predetermined concentration of the chemical tested dissolved in water. Luminescence and fluorescence in the microtiter plates were measured using an Infinite M200 PRO plate reader (Tecan) at 37°C, at 10-min intervals. Each assay was repeated at least three times. Fluorescence and luminescence values are presented as the instrument’s arbitrary relative fluorescence and luminescence units (RFU and RLU, respectively). Activity is displayed as the difference in the intensity of the signal in the presence and absence of the inducer (ΔRFU and ΔRLU). The effect of a mutation on reporter induction is displayed as the mutation effect, calculated as follows:

$$Mutation\,\;effect=\frac{{Max}_{0\to 300\,\;\min}\,\;\left({\Delta RLU}_{Mu\,\tan t}\right)}{{Max}_{0\to 300\,\;\min}\;({\Delta RLU}_{Wild\,\;type})}*100-100$$

Max_0→300_ denotes the maximal ΔRFU or ΔRLU value obtained in the course of a 300 min exposure of the reporter cells to the tested inducer.

The effect of a gene overexpression on bioreporter induction was calculated in the same manner, using the ΔRFU values of the strain harboring the overexpression plasmid instead of the ΔRLU of the mutant in the equation above.

### DNT Degradation Assay

The tested strains (*ΔyhaK, ΔyhaJ* and the wild type, BW25113) were cultured in TGA overnight and regrown as previously described. DNT was added to a concentration of 50 mg/L and the bacteria were incubated at 37°C, 200 rpm. Spent medium samples were collected at 0, 240, 300, 360, 420 and 480 min, and tested for the presence of the *yqjF* inducer in a luminescence assay as described above, using the *yqjF::luxCDABE*-harboring reporter strain.

## Results

### Identification of a Regulatory Gene Affecting the Induction of the *yqjF* Gene Promoter by 2,4-DNT

**FIGURE 1:**
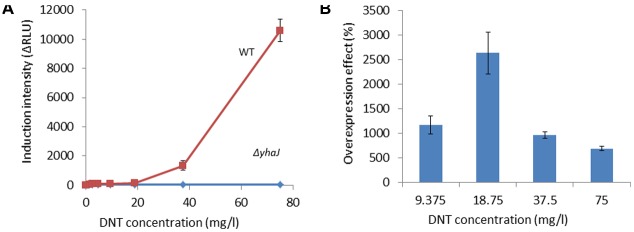
**Induction of *yqjF* by DNT is abolished in a *yhaJ* mutant and enhanced when YhaJ is overexpressed.** **(A)** Induction intensity (maximal luminescence recorded within 300 min of exposure) of *yqjF* as a function of DNT concentration in a *ΔyhaJ* mutant and the WT. **(B)** Effect of overexpressed YhaJ on *yqjF* induction by several DNT concentrations. Calculation of the overexpression effect is as detailed in Section “Materials and Methods.” Error bars represent the standard deviation of at least three independent duplicate experiments.

As no regulatory pathway of DNT degradation in *E. coli* has been described to date, nor has any regulatory element involved in *yqjF* activation been reported, we have performed a broad scan of regulatory gene mutations for their effect on *yqjF* induction. A plasmid harboring a fusion of the *yqjF* gene promoter to the *Photorhabdus luminescens luxCDABE* gene cassette (*yqjFwt::lux*, **Table 1**) was used to transform 150 Keio collection clones, each with a single regulatory gene deletion (**Supplementary Table S2**), and the intensity of luminescent response to DNT was measured. To verify the specificity of the effect on *yqjF*, mutants found to affect *yqjF* induction were also tested for their effect on *lacZ* induction by isopropyl β-D-1-thiogalactopyranoside (IPTG) and on *recA* induction by nalidixic acid (NA), using plasmids pBR2TTS*:lacZ::luxCDABE* and pBR2TTS:*recA::luxCDABE*, respectively. Out of the 150 genes tested, only the *yhaJ* mutant was found to totally inhibit the induction of *yqjF* by DNT at all DNT concentrations tested (**Figure 1A**). The same mutation also completely inhibited *yqjF* induction by other known inducers of this gene: TNT, hydroquinone, 2-metoxy-5-nitroaniline, 1,2,4-trihydroxybenzene and catechol (**Supplementary Table S3**).

To further verify the role of YhaJ in the regulation of y*qjF* induction by 2,4-DNT, the *yhaJ* gene was overexpressed by inserting it along with its own promoter into the pBR2TTS:*yqjF::luxCDABE* plasmid. When *yqjF* induction by DNT in an *E. coli* strain harboring the YhaJ-overexpressing plasmid was compared to that of the same strain harboring the original plasmid, a significant enhancement was observed in the intensity of the response. This may be observed in **Figure 1B**, which displays the overexpression effect, which displayed a value of ca. 3,000% (implying a 30-fold signal intensity enhancement) at a DNT concentration of 18.75 mg/l.

### Identification of a YhaJ Regulatory Binding Motif

YhaJ has recently been identified in *E. coli* O157:H7 as a functional LTTR that has been adapted to regulate the virulence of this pathotype (Connolly et al., 2016). However, data described in the present study suggest additional and diverse roles for this transcription factor. To identify additional promoters under the regulation of YhaJ in the context described here, and investigate whether they share a common binding motif, we have searched for additional genes induced by hydroquinone, a compound previously shown (Yagur-Kroll et al., 2014) to directly induce *yqjF*. This was carried out by exposing to hydroquinone a library consisting of transcriptional fusions of GFP to approximately 2,000 different *E. coli* K12 promoters (Zaslaver et al., 2006). Seventeen gene promoters were activated to different degrees, out of which eight were selected for further investigation based upon the kinetics of the reaction (low background and rapid response time) and its intensity (**Table 2**).

**Table 2:** Gene promoters induced by hydroquinone, categorized by induction intensity.

Induction category: induction intensity (ΔRFU):	Weak (200–600)	Intermediate (601–1100)	Strong >1100
	*ydjN*	*ygiD^∗^*	*yhaK^∗^*
	*ddlA*	*ycfR^∗^*	*rplY*
	*nrdD*	*manX*	*Asr*
	*cysD*	*dsrA*	*yceP^∗^*
		*rsd*	*ybiJ^∗^*
		*yeeE^∗^*	
		*yhhW^∗^*	


*Gene promoters selected for further investigation are marked by an asterisk.*

**FIGURE 2:**
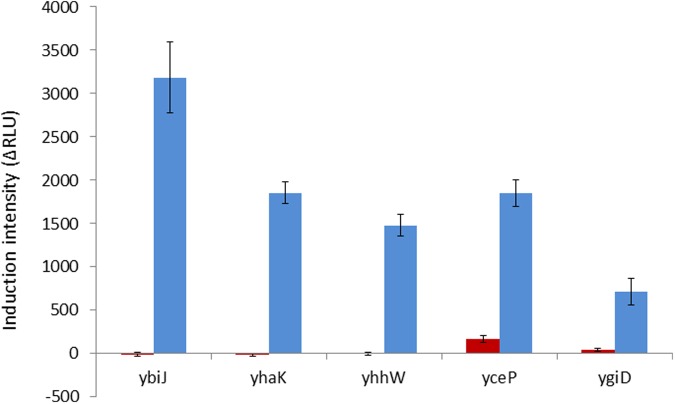
**Induction intensity (maximal luminescence recorded within 300 min of exposure) by hydroquinone (75 mg/l) of the five gene promoters found to be affected by the lack of YhaJ.** Red bars, plasmids harboring the promoter:gfp fusion in a ΔyhaJ background; blue bars, same plasmids in the WT host. Error bars represent the standard deviation of at least three independent duplicate experiments.

When the activation of these eight gene promoters by hydroquinone was then tested in a *ΔyhaJ* background, five of them were found to be negatively affected by the lack of YhaJ – the induction of *yhaK, ybiJ, ygiD* and *yhhw* was entirely eliminated, and that of *yceP* was greatly reduced (**Figure 2**).

**FIGURE 3:**
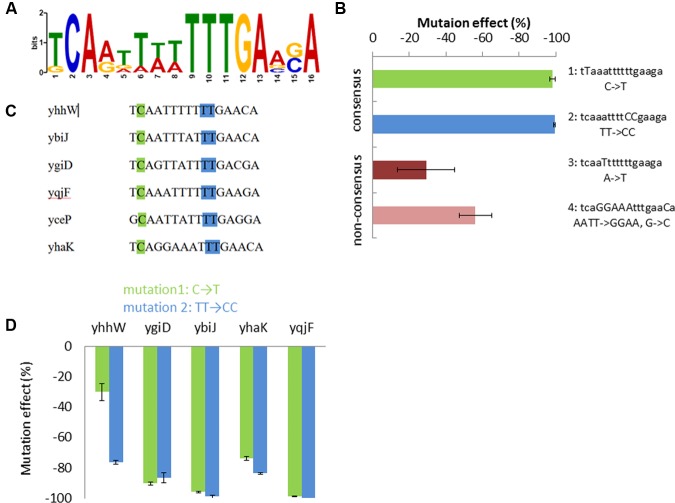
**Proposed YhaJ binding motif.** **(A)** The significant common motif found by MEME; **(B)** effect of the four mutated sequences of the *yqjF* promoter on its induction by DNT (35 mg/l); **(C)** the motif sequence in the different YhaJ-regulated promoters; mutation sites 1 (green) and 2 (blue) are marked; **(D)** the effect of mutations sets 1 and 2, when introduced into YhaJ-regulated gene promoters, on their induction by hydroquinone (75 mg/l). Error bars represent the standard deviation of at least three independent duplicate experiments.

To search for a possible common YhaJ binding motif in the promoters affected by its absence, the intergenic regions before each gene were considered as promoter sequences. These were extracted from EcoGene (Rudd, 2000) and analyzed in MEME (Bailey et al., 2006). A motif of 16 bases was identified, with an E-value of 4.0e-003 (**Figure 3A**), displaying a similarity to that of the “typical” LTTR binding sequence – T-N11-A. No significant common motifs were found when the same program was used to search all gene promoters previously found to be induced by DNT (Yagur-Kroll et al., 2014) and the additional gene promoters induced by hydroquinone (**Table 2**).

### Effects of Mutations in the Motif Sequence

To investigate the identified motif, four mutations were inserted into the *yqjF* promoter sequence of the *yqjF::luxCDABE* plasmid (**Figure 3B**), and strains containing these modified plasmids were tested for induction by DNT. Two of the introduced mutations (mutations 1 and 2) affected the consensus sequence of the motif, mutation 3 modified a base which is not conserved in all of the promoters tested, and in mutation 4 six such bases were modified to render the sequence in the *yqjF* promoter identical to that found in *yhaK* (**Figure 3B**). This last change was implemented in order to check if the response to DNT will be substantially reduced, as *yhaK* was seen to display a very low induction by DNT in WT cells. As observed in **Figure 3B**, mutations in the consensus sequence (1 and 2) have completely obliterated the induction of *yqjF* by DNT; mutation 3 caused only a minor inhibition, while mutation 4 yielded a decrease in activity by over 50%.

To examine whether the consensus sequence was also important for the induction of the other genes found to be affected by *ΔyhaJ*, mutations 1 and 2 were also inserted into the promoters of four of these genes (*yhaK, ybiJ, ygiD*, and *yhhW*). As shown in **Figure 3D**, these mutations caused a significant decrease in induction by hydroquinone in all tested promoters.

### YhaJ Directly Regulates DNT-Inducible Genes

**FIGURE 4:**
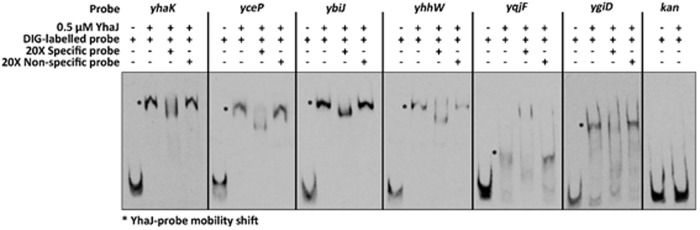
**Electrophoretic mobility shift assay (EMSA) analysis of gene promoter regions induced by exposure to DNT.** DIG-labeled DNA probes corresponding to the promoter regions of *yhaK, yceP, ybiJ, yhhW, yqjF*, and *ygiD* were incubated with 0.5 uM of purified YhaJ per reaction. The presence of YhaJ resulted in an observable mobility shift (^∗^) when compared to the probe only controls. A fragment of the *kan* gene was used a non-specific control to indicate no binding. Competition assays were also performed with a 20-fold excess of unlabeled specific or non-specific probe. The specific competitors were able to reduce the intensity of band shift observed for YhaJ whereas addition of the *kan* probe had no effect on the band shift observed. The probe names and composition of each reaction is indicated at the top of the figure with a – or + indicating absence or presence of that component respectively.

The ability of YhaJ to bind to the six gene promoters was tested by an EMSA. Upstream regions (approximately 300 bp in length) of the six gene coding sequences described above were used as probes for purified YhaJ in the binding reaction. The data (**Figure 4**) clearly show that *in vitro*, YhaJ is capable of binding to the six promoter regions suspected to be under its regulation. This is indicated by retardation in the band running position when compared to free DNA alone and is suggestive of a protein-DNA interaction. To address the specificity of these band shifts, competition assays were performed using a 20-fold excess of both unlabeled specific probe and unlabeled non-specific probe. A ∼350 bp fragment of the kanamycin gene from pUC57 was used as a non-specific probe and as a negative control for YhaJ binding. In the competition assays, an excess of specific probe in each reaction caused a drop in the band shift pattern, suggesting competition for the labeled probe and a reduction in band intensity. Conversely, the non-specific competitor had no effect on the band shift pattern in all reactions tested. Taken together, these results indicate that YhaJ has the capacity to directly bind the promoter regions of genes identified as being part of its DNT regulon.

### Induction of *yqjF* by DNT Is Enhanced in a *yhaK* Mutant

**FIGURE 5:**
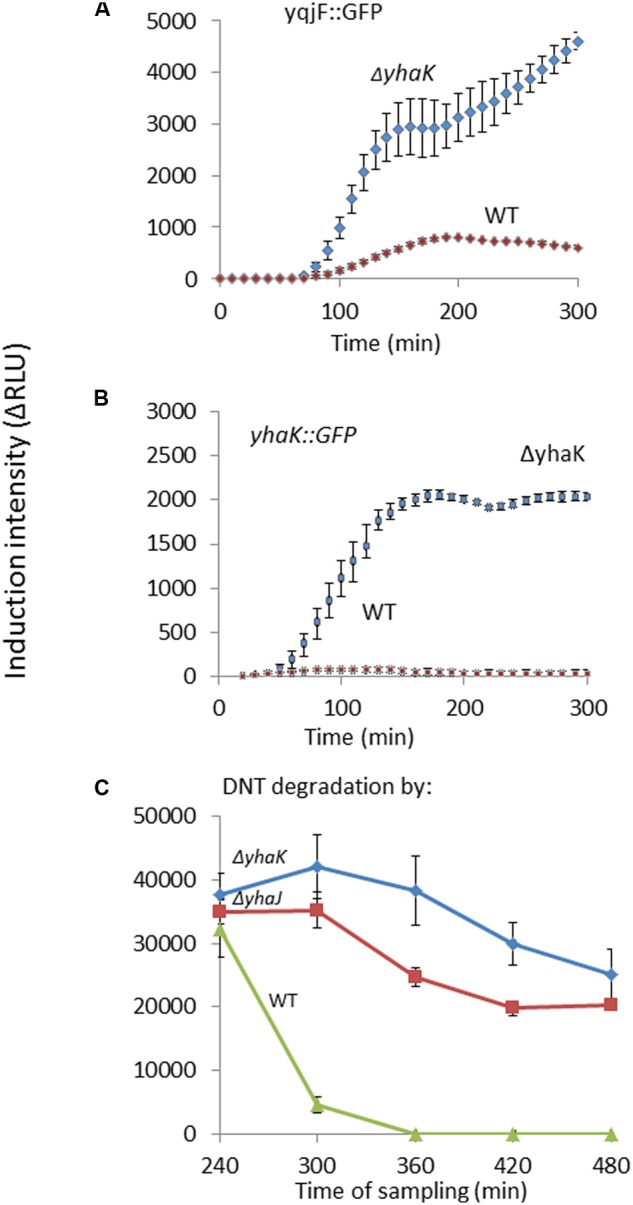
**Role of *yhaK* in DNT degradation and *yqjF* induction.** Effect of a *ΔyhaK* mutation on **(A)** yqjF and **(B)** *yhaK* induction by DNT (75 mg/l) as a function of time. **(C)** Accumulation of the *yqjF* inducing molecule in *ΔyhaK* and *ΔyhaJ* mutants. Error bars represent the standard deviation of at least three independent duplicate experiments.

The induction of *yqjF::GFP* was also tested in host strains mutated in the genes demonstrated above to be regulated by YhaJ. The *ΔyhaK* mutant had a dramatically enhanced effect on *yqjF* induction by DNT (**Figure 5A**); similar results were obtained for other known *yqjF* inducers, hydroquinone and catechol (**Supplementary Table S4**). To understand the importance of *yhaK* in the cellular reaction to DNT we also tested its own response to DNT in the same *ΔyhaK* mutant. Similarly to *yqjF*, a much stronger induction was observed in the mutant compared to the WT (**Figure 5B**). One possible explanation for this phenomenon is that the *yhaK* product is also a transcription factor, as earlier suggested by Gurmu et al. (2009). An additional (and not mutually exclusive) scenario is that this gene encodes an enzyme involved in the degradation of the yet unidentified inducer molecule, itself a degradation product of DNT, and that in its absence an accumulation of this molecule leads to stronger induction of both *yqjF* and *yhaK*. To test this hypothesis we monitored *yqjF* induction intensity when exposed to samples collected during 8 h of DNT degradation by cultures of the *ΔyhaK* and *ΔyhaJ* mutants, in comparison to their WT. The results (**Figure 5C**) show that *yqjF*-inducing activity almost completely disappears from the WT medium after ca. 300 min, whereas in media in which DNT was degraded by the mutants, this activity remains high even after 480 min.

## Discussion

An *E. coli*-based bioreporter for the detection of trace amounts of 2,4-dinitrotoluene and 2,4,6-trinitrotoluene, both signature chemicals of buried landmines, was previously described by Yagur-Kroll et al. (2014). The DNT-inducible *yqjF* gene promoter successfully served as the sensor element of this construct, but the regulatory mechanism controlling its activation has remained undeciphered. In the study reported herein we have discovered the main player involved in this regulation, the protein YhaJ, and have shed light on some aspects of this regulation.

### YhaJ Is the Transcription Factor of yqjF

YhaJ, a member of the LysR protein family, was recently discovered by Connolly et al. (2016), who described a dual function of this protein – regulation of both D-serine uptake by a novel mechanism and the locus of an enterocyte effacement (LEE) island, a horizontally acquired pathogenicity element, in enterohemorrhagic *E. coli* O157:H7. It was found that YhaJ directly regulates the expression of the *dlsT* (*yhaO*) transporter of D-serine under conditions promoting virulence, and that of the *ler* gene, the master regulator of the LEE island (Connolly et al., 2016). The same group had also previously linked D-serine sensing with virulence in *E. coli* O157:H7 (Connolly et al., 2015). However, it was proposed that this was a rather adapted role for YhaJ and that other functions for this highly conserved regulator likely exist due to a wider YhaJ regulon being identified by RNA-seq analysis.

In the present study, a knockout mutant of *yhaJ* was found to completely erase the response of *yqjF* to DNT and to other known inducers, while overexpression of YhaJ improved the sensitivity and intensity of *yqjF*’s induction by DNT. When a plasmid-borne *yhaJ* gene was expressed in the *ΔyhaJ* strain, the induction of *yqjF* was restored. We thus suggest that YhaJ is the transcriptional regulator of *yqjF*. This hypothesis is bolstered by RNA-seq data recently described for *ΔyhaJ* in *E. coli* O157:H7 that revealed *yqjF* as being differentially expressed with high significance in the *ΔyhaJ* mutant background (Connolly et al., 2016). As the yet unidentified inducer of *yqjF* accumulates in a *ΔyhaJ* mutant, we conclude that this regulation directly affects the degradation of the inducer molecule rather than its formation.

### Five Additional Genes Are Regulated by YhaJ

In the course of this study, we have also identified five additional gene promoters regulated by YhaJ and have verified their binding by EMSA. While over a dozen LysR family proteins from different bacterial species have been linked to aromatic compound degradation, none of them was shown to regulate more than three gene promoters (with most of them regulating only one; Junker et al., 1997; Tropel and Van Der Meer, 2004; Kubota et al., 2014). YhaJ thus appears to be unique in its ability to regulate at least six genes found on different locations on the chromosome. One of the genes shown here to be under YhaJ regulation, *ybiJ*, has been previously suggested as an additional bioreporter candidate for the detection of DNT (Yagur-Kroll et al., 2014). Interestingly, *ybiJ* and *yhaK* have been previously found to be induced by nitrite (NO_2_^-^) (Mukhopadhyay et al., 2004). The fact that both *ybiJ* and *yhaK* are induced upon DNT exposure may indicate that nitrite groups may be released from DNT in the course of its degradation.

### The YhaJ Binding Motif

Most LTTRs have two binding domains in the regulated promoter, the ribosome binding site (RBS) and the activator binding site (ABS; Maddocks and Oyston, 2008). The RBS contains a general LTTR-box with the sequence T-N_11_-A; often this sequence is in the form found in the common motif of five YhaJ-regulated genes, *yqjF, ybiJ, yhaK*, *ygiD*, and *yhhW* (**Figure 3**), where there is evidence of an inverted repeat sequence, TCA-N_7_-TGA. The G-N_11_-A sequence found in *yceP* that breaks the inverted repeat was also observed as an imperfect LTTR box in *catBC* promoter regulated by CatR. In this case, a directed mutation (G72T) improved binding to and activation of the promoter (Parsek et al., 1994).

We suggest herein that the motif presented in **Figure 3A** is the RBS of YhaJ. This claim is supported by the fact that mutations introduced into the motif sequence of the *yqjF* gene promoter caused a reduction in the induction by DNT. While a 6-base change in non-consensus bases caused a maximal reduction of 60%, a change of only one nucleotide from the consensus bases in the sequence caused a 98% decrease in induction. After random mutagenesis of the *yqjF* promoter region conducted by Yagur-Kroll et al. (2015), the 13th base of this motif was changed from A to G, causing a completely inhibition of *yqjF* induction by DNT. Mutations in the consensus sequence of other promoters harboring this motif showed a substantial negative effect also on induction by hydroquinone.

### A Suggested Role for YhaJ in *E. coli*

Not much is known about the functional role of the genes regulated by YhaJ in *E. coli*. YhaJ has been recently shown to regulate virulence in pathogenic *E. coli*, but LTTRs often have multiple and diverse roles in the cell’s metabolism as well as in its virulence (Maddocks and Oyston, 2008; Connolly et al., 2016). The structures of YhaK, YgiD, and Yhhw have been determined, and possible enzymatic activities have been suggested. YgiD is a close homolog of known structure for the plant enzyme 4,5-DOPA-extradioldioxygenase, which catalyzes the transformation of LL-DOPA to 4,5-seco-DOPA. *In vitro* experiments have shown that YgiD also possesses this activity, and is able to transform L-DOPA to betalamic acid and muscaflavin (Gandía-Herrero and García-Carmona, 2014). YhhW is a pirin protein which was found to possess quercetinase activity, releasing carbon monoxide as a reaction by-product (Adams and Jia, 2005).

As mentioned above, many LTTRs are known to be regulators of aromatic compound degradation. We suggest that YhaJ is a regulator of the degradation of quinol (and related compounds) in *E. coli*. This is further supported by the fact mentioned above that both YhhW and YgiD, which are regulated by YhaJ, are able to cleave quinol-like aromatic structures.

### YhaK Plays a Role in the Biotransformation of DNT

YhaK is an *E. coli* protein of a yet unknown function. Its structure was determined by Gurmu et al. (2009), who found that *in vitro* it is a good marker for monitoring oxidative stress in *E. coli*. They have also suggested that YhaK is a transcriptional co-factor of YhaJ, due to its similarity (13%) to the human hPirin which is found only in the nucleolus and is involved in DNA transcription (Gurmu et al., 2009). In the work reported here, we have found that *yhaK* is regulated by YhaJ, its divergently transcribed gene. We have also discovered that the *yhaK* promoter is induced by DNT and by hydroquinone (or their metabolites). As *yqjF* induction by DNT in a *ΔyhaK* mutant was 66-fold higher than in the WT, we suggest that YhaK plays a role in the degradation of DNT. To further support this argument we have shown that DNT degradation products from a *ΔyhaK* strain contain the *yqjF* inducer even after 480 min of degradation, indicating that the inducer accumulates in this mutant and is not degraded as fast as in the WT. We thus suggest that in addition to its potential role as a transcription factor, YhaK also functions as an enzyme involved in the degradation of quinol and quinol-like compounds.

## Author Contributions

SB and NP designed the experiments; NP carried out most of the experimental work, assisted by BS. JC cloned and purified YhaJ, carried out the EMSA experiment and analyzed its results. NP, JC, and SB wrote the article.

## Conflict of Interest Statement

The authors declare that the research was conducted in the absence of any commercial or financial relationships that could be construed as a potential conflict of interest.
