# Supplementary material for: The Highly Conserved Escherichia coli Transcription Factor YhaJ Regulates Aromatic Compound Degradation
Source: Front Microbiol. 2016 Sep 22;7:1490. doi: 10.3389/fmicb.2016.01490 (PMC5031710; doi:10.3389/fmicb.2016.01490)
Supplement: Supplementary file 1 [file Table_1.DOCX]

**SUPPLEMENTARY MATERIALS**

### The highly conserved *Escherichia coli* transcription factor YhaJ regulates aromatic compound degradation

Noa Palevsky^1^, Benjamin Shemer^1^, James PR Connolly^2^ and Shimshon Belkin^1^*

^1^Institute of Life Sciences, the Hebrew University of Jerusalem, Jerusalem 91904, Israel

^2^College of Medical, Veterinary and Life Sciences, University of Glasgow, 120 University Place, Glasgow G12 8TA United Kingdom

Running title: YhaJ regulates aromatic compound degradation in *E. coli*

* Corresponding author at [shimshon.belkin@mail.huji.ac.il](mailto:shimshon.belkin@mail.huji.ac.il)

**Table S1.** PCR primers used in this work

|  | **Primer sequence** |  |
| --- | --- | --- |
|  | Mutations in the motif site of *yqjF* |  |
| Mut1-F | CTTATCCCTTAAATTTTTTGAAGATTTTTGACA | This work |
| Mut1-R | TGTCAAAAATCTTCAAAAAATTTAAGGGATAAG | This work |
| Mut2-F | CTTATCCCTCAAATTTTCCGAAGATTTTTGACA | This work |
| Mut2-R | TGTCAAAAATCTTCGGAAAATTTGAGGGATAAG | This work |
| Mut3-F | CCTTATCCCTCAATTTTTTTGAAGATTTTTGAC | This work |
| Mut3-R | GTCAAAAATCTTCAAAAAAATTGAGGGATAAGG | This work |
| Mut4-F | CGGCCTTATCCCTCAGGAAATTTGAACATTTTTGACA | This work |
| Mut4-R | TGTCAAAAATGTTCAAATTTCCTGAGGGATAAGGCCG | This work |
| yqjF-F | GGTGGTACCCGGTTTTGGCGTATGG | yagor |
| YqjF-R | CGGAGCTCGCCACTCAGGCTGCTGAT | This work |
|  | Mutations in the motif site of *yhaK* |  |
| yhaK-F | CACCGCTCGAGACGCGTCCCAGCTCATC | This work |
| Yhak-R | CACCGGGATCCGCCTGCAACCATCCGTAG | This work |
| Yhak-mut1-F | CCATTTCGTTCTCATTTAGGAAATTTGAACA | This work |
| Yhak-mut1-R | TGTTCAAATTTCCTAAATGAGAACGAAATGG | This work |
| yhaK-mut2-F | CATTCAGGAAATCCGAACATACCGGGCAG | This work |
| yhaK-mut2-R | CTGCCCGGTATGTTCGGATTTCCTGAATG | This work |
|  | Mutations in the motif site of *yhhw* |  |
| yhhW-F | CACCGCTCGAGTCGGATCGTAATAGTTGGCA | This work |
| yhhW-R | CACCGGGATCCGAGCAAGCCTCTCCCTCC | This work |
| yhhW-mut1-F | CCCCTCGAATAGTTTAATTTTTTTGAACAGA | This work |
| yhhW-mut1-R | TCTGTTCAAAAAAATTAAACTATTCGAGGGG | This work |
| yhhW-mut2-F | CGAATAGTTCAATTTTTCCGAACAGAGGGGTC | This work |
| yhhW-mut2-R | GACCCCTCTGTTCGGAAAAATTGAACTATTCG | This work |
|  | Mutations in the motif site of *ybiJ* |  |
| ybiJ-F | CACCGCTCGAGGTTCTGTGCCTGGGATGC | This work |
| ybiJ-R | CACCGGGATCCCGCAGAGGTTCGAAAGACA | This work |
| ybiJ-mut1-F | GGTTAGATATCAATTAATTTATTTGAACAAG | This work |
| ybiJ-mut1-R | CTTGTTCAAATAAATTAATTGATATCTAACC | This work |
| ybiJ-mut2-F | CAATCAATTTATCCGAACAAGGCGGTCAATTC | This work |
| ybiJ-mut2-R | GAATTGACCGCCTTGTTCGGATAAATTGATTG | This work |
|  | Mutations in the motif site of *ygiD* |  |
| ygiD-F | CACCGCTCGAGCGTTCATCGGACTACCGTG | This work |
| ygiD-R | CACCGGGATCCCAATAAACGTGGCTGCCC | This work |
| ygiD-mut1-F | GATCGTCAAAATAACTAATGATCATCAT | This work |
| ygiD-mut1-R | ATGATGATCATTAGTTATTTTGACGATC | This work |
| ygiD-mut2-F | CCTTCAGGCAGATCGTCGGAATAACTGATG | This work |
| ygiD-mut2-R | CATCAGTTATTCCGACGATCTGCCTGAAGG | This work |
| Gfp-seq3 | GATCTGGGTATCTCGCAAAGC | This work |
|  | *yhaJ:yhaJ* into *yqjF:lux* |  |
| yhaJ-P-F | GCTACGAATTCCGGGTCGAAGTAGTGTCCA | This work |
| yhaJ-P-R | GTAGCGAATTCTCTTAGAATTGGGGCGATATTT | This work |
|  | *yhaJ-phis* |  |
| yhaJ- F – his | GGATCCATGGCCAAAGAAAGGGCATT | Connolly *et al.*  (2016) |
| yhaJ-R- his | AAGCTTTTATTTTCCGTTAAAAAGTT | Connolly *et al.*  (2016) |
|  | Sequences used for amplifying promoter regions for EMSA |  |
| yhaK F | CATTGGTGAATTTGGTAC | This work |
| yhaK R | GCAGTTCGGGTAGTAATCAT | This work |
| yceP F | ACTGGTTTGTCAGCGAATAA | This work |
| Ycep R | TCTGAATGACTTCATTATTTTT | This work |
| ybiJ F | GATATAATCGCAGAGGTTCG | This work |
| ybiJ R | AGCAGCAACAACAGTATTGA | This work |
| yhhW F | CGGTCAGAGAAGAGATGAAG | This work |
| yhhW R | TCATTTGCTTTGCGTAAGTA | This work |
| yqjF F | TGGATCTTTCCGCCAGTC | This work |
| yqjF R | CATAAACGTGGTGAATGATG | This work |
| ygiD F | ACATTCCTTCAGCTGCTAGT | This work |
| ygiD R | AAATGACACCTTTAGTTAAGGA | This work |
| Kan F | GGCAAAAGTTTATGCATTTC | This work |
| Kan R | GCATTTTATCCGTACTCCTG | This work |
|  |  |  |

**Table S2.** The Kieo collection *E. coli* mutant strains (Baba et al., 2006) tested for their effect on *yqjF* induction by DNT

| **Strain** | **Deletion** | **Strain** | **Deletion** | **Strain** | **Deletion** |
| --- | --- | --- | --- | --- | --- |
| JW0063 | ∆araC | JW1612 | ∆malI | JW3524 | ∆yiaG |
| JW0075 | ∆leuO | JW1686 | ∆ydiP | JW3546 | ∆yiaJ |
| JW0109 | ΔpdhR | JW1728 | ∆osmE | JW3557 | ∆yiaU |
| JW0141 | ∆dksA | JW1779 | ∆yeaM | JW3644 | ∆uhpA |
| JW0198 | ΔyafC | JW1788 | ∆yeaT | JW3656 | ∆yidL |
| JW0230 | ∆crl | JW1880 | ∆flhC | JW3721 | ΔasnC |
| JW0265 | ∆ yag | JW1881 | ∆flhD | JW3746 | ΔilvY |
| JW0298 | ∆ykgD | JW1899 | ∆uvrY | JW3755 | ∆rho |
| JW0322 | ∆prpR | JW1907 | ∆fliA | JW3804 | ∆metR |
| JW0336 | ∆lacI | JW1935 | ∆rcsA | JW3818 | ∆rfaH |
| JW0337 | ΔmphR | JW1967 | Δnac, | JW3839 | ∆glnG |
| JW0389 | ∆phoB | JW2074 | ∆gatR | JW3840 | ∆glnL |
| JW0390 | ∆phoR | JW2138 | ΔgalS | JW3841 | ∆glnA |
| JW0437 | ∆ybaO | JW2144 | ∆yeiE | JW3843 | ∆yihL |
| JW0440 | ∆glnK | JW2201 | Δada | JW3876 | ∆rhaS |
| JW0476 | ∆cueR | JW2361 | ∆dsdC | JW3905 | ΔcytR |
| JW0494 | ∆allR | JW2366 | ∆evgA | JW3909 | ΔmetJ |
| JW0555 | ∆envY | JW2393 | ∆yfeC | JW3926 | ∆yijO |
| JW0560 | ∆cusR | JW2476 | ∆hyfR | JW3933 | ∆oxyR |
| JW0602 | ∆rnk | JW2537 | ∆glnB | JW3959 | ∆rsd |
| JW0624 | ΔybeF | JW2561 | ∆yfiE | JW4023 | ΔsoxS |
| JW0669 | ∆fur | JW2659 | ∆mprA | JW4024 | ΔsoxR |
| JW0719 | ∆mngR | JW2675 | ∆gutM | JW4050 | ∆rpiR |
| JW0780 | ∆ybiH | JW2676 | ΔsrlR | JW4085 | ∆dcuR |
| JW0824 | ΔdeoR | JW2701 | ∆fhlA | JW4136 | ∆yjeB |
| JW0883 | ∆ycaN | JW2705 | ∆ygbI | JW4149 | ∆ulaR |
| JW0895 | ∆ihfB | JW2755 | ∆relA | JW4253 | ∆fecI |
| JW0941 | ∆sulA | JW2776 | ΔfucR | JW4261 | ∆yjhI |
| JW0980 | ∆torR | JW2779 | ∆gcvA | JW4287 | ΔuxuR |
| JW0998 | ∆ ycd | JW2805 | ΔgalR | JW4356 | ΔtrpR |
| JW1116 | ∆phoP | JW2807 | ΔlysR | JW4359 | ∆rob |
| JW1149 | ∆ycgE | JW2883 | ΔargP | JW4361 | ∆creB |
| JW1208 | ∆chaB | JW2947 | ∆glcC | JW5060 | ∆bolA |
| JW1213 | ∆narX | JW2985 | ∆sufI | JW5114 | ∆ybjK |
| JW1267 | ∆cysB | JW2993 | ∆qseB | JW5135 | ∆torS |
| JW1296 | ∆pspF | JW3032 | ∆ygiP | JW5248 | ∆marR |
| JW1321 | ∆ycjZ | JW3039 | ∆rpoD | JW5267 | ∆slyA |
| JW1379 | ∆feaR | JW3065 | ∆exuR | JW5322 | ∆yedW |
| JW1394 | ∆paaX | JW3073 | ∆yqjG | JW5406 | ∆yphH |
| JW1434 | ∆ydcR | JW3076 | ∆yhaJ | JW5434 | ∆ascG |
| JW1445 | ∆ ync | JW3077 | ∆yhak | JW5437 | ∆rpoS |
| JW1494 | ∆ yde | JW3100 | ∆agaR | JW5476 | ∆ ygf |
| JW1501 | ∆hipB | JW3157 | ∆yrbA | JW5517 | ∆yhaL |
| JW1505 | ∆ydeW | JW3169 | ∆rpoN | JW5607 | ∆hdfR |
| JW1519 | ∆yneJ | JW3188 | ∆yhcF | JW5702 | Δcrp, |
| JW1533 | ∆ydfH | JW3195 | ∆nanR | JW5703 | ∆yheO |
| JW1556 | ∆relB | JW3212 | ∆aaeR | JW5843 | ∆norR |
| JW1566 | ∆dicB | JW3368 | ∆ompR | JW5894 | ∆cynR |
| JW1586 | ∆dgsA | JW3483 | ∆gadW | JW5908 | ∆yddM |
| JW1600 | ∆rstA | JW3484 | ∆gadX | JW5946 | ∆gntR |

**Table S3** A *yhaJ* mutation inhibits *yqjF* induction (assayed by *yqjF::luxCDABE* luminescence) by known inducers of this gene

| Compound (mg/L) | Luminescence (ΔRLU) in the WT | Luminescence (ΔRLU) in *ΔyhaJ* | % inhibition |
| --- | --- | --- | --- |
| Catechol (200) | 751,350 (±7700) | 280 (±110) | 99.6 (±$0.08)$ |
| Hydroquinone (200) | 3,117,640 (±266,000) | 2,940 (±560) | 99.9 ($\pm0.2$) |
| 1,2,4-trihydroxybenzene (60) | 197,090 (±5,780) | 170 (±18) | 99.2 ($\boldsymbol{\pm0.06}$) |
| 2,4,6-trinitrotoluene (100) | 16,640 (±1,470) | 40 (±7) | 99.8 ($\pm0.2$) |
| 2-metoxy-5-nitroaniline (80) | 1,900 (±430) | 15 (±4) | 99.9 ($\pm0.3$) |

Wild-type *E. coli* strain BW25113 and a *yhaJ*-deficient mutant were transformed with plasmid pBR2TTS:*yqjF::luxCDABE*, and their bioluminescent responses to the presence of 5 different chemicals were monitored for 9 h. The values in this table represent the average response (± standard deviation) 180 min after addition of the tested chemicals, in three independent experiments. ΔRLU: the difference in luminescence in the presence and the absence of the inducer, in the microtiter plate reader’s (Infinite M200 PRO, Tecan) arbitrary relative light units.

**Table S4** A *ΔyhaK* mutation enhances *yqjF* induction (assayed by *yqjF::luxCDABE* luminescence) by hydroquinone and catechol

| Compound | Luminescence (ΔRLU) in the WT | Luminescence (ΔRLU) in *ΔyhaK* | Mutation effect (%) |
| --- | --- | --- | --- |
| Catechol | 415,000 (±47,000) | 1,320,000 (±82,000) | 218.2 (+/-20.5) |
| Hydroquinone | 563,000 (±59,000) | 2,380,000 (±73,000) | 327.8 (+/-54.0) |

Wild-type *E. coli* strain BW25113 and a *yhaK*-deficient mutant were transformed with plasmid pBR2TTS:*yqjF::luxCDABE*, and their bioluminescent responses to catechol and hydroquinine (both at 250 mg/L) were monitored for 9 h. The values in this table represent the average response (± standard deviation) 180 min after addition of the tested chemicals, in three independent experiments. Mutation effect was calculated as $\frac{\left( {\Delta RLU}_{Mutant} \right) at 180 min}{\left( \Delta{RLU}_{Wild type} \right) at 180 min}*100-100$. ΔRLU: the difference in luminescence in the presence and the absence of the inducer, in the microtiter plate reader’s (Infinite M200 PRO, Tecan) arbitrary relative light units.

**References:**

Baba, T., Ara, T., Hasegawa, M., Takai, Y., Okumura, Y., Baba, M., et al*.* (2006). Construction of *Escherichia coli* K‐12 in‐frame, single‐gene knockout mutants: the Keio collection. *Mol. Syst. Biol*. 2. doi: [10.1038/msb4100050](http://dx.doi.org/10.1038/msb4100050)

Connolly, J.P.R, Gabrielsen, M., Goldstone, R.J., Grinter, R., Wang, D., Cogdell, R.J,et al. (2016) A highly conserved bacterial D-serine uptake system links host metabolism and virulence. *PLoS Pathog* 12(1): e1005359 doi: [10.1371/journal.ppat.1005359](http://dx.doi.org/10.1371/journal.ppat.1005359)
